# Supplementary figures and images for: Internalization of Salmonella in Leafy Vegetables during Postharvest Conditions
Source: Foods. 2023 Aug 18;12(16):3106. doi: 10.3390/foods12163106 (PMC10453844; doi:10.3390/foods12163106)

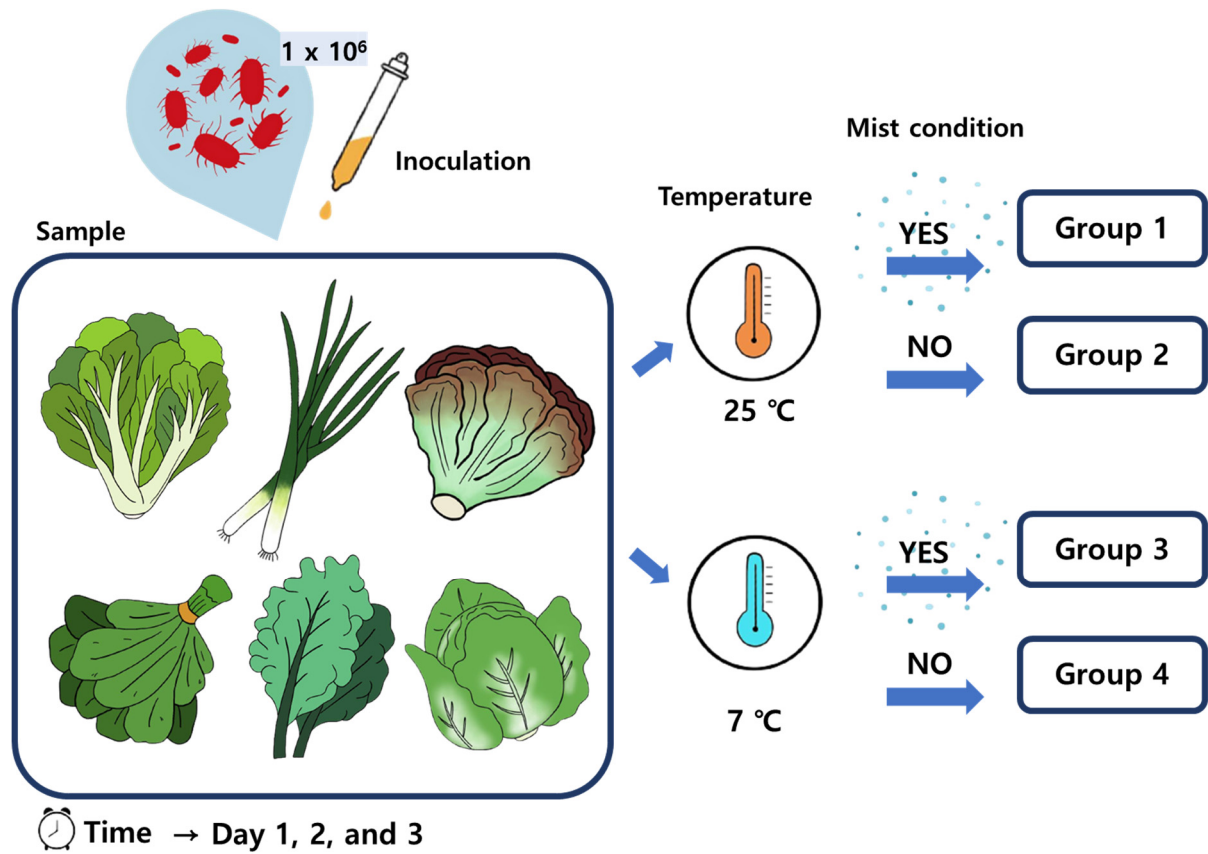

**Figure S1.** Summary of the study design

Supplement: Supplementary file 1 [file foods-12-03106-s001.zip › foods-2531144-supplementary.pdf]
